# Ranking-space: magnitude makes sense through spatially scaffolded ranking

**DOI:** 10.3389/fpsyg.2023.1224254

**Published:** 2023-07-06

**Authors:** Elger Abrahamse, Jean-Philippe van Dijck

**Affiliations:** ^1^Department of Communication and Cognition, Tilburg University, Tilburg, Netherlands; ^2^Department of Educational Sciences, Atlántico Medio University, Las Palmas, Spain; ^3^Expertise Centre for Care and Welfare, Thomas More, Antwerp, Belgium; ^4^Department of Experimental Psychology, Ghent University, Ghent, Belgium

**Keywords:** numerical cognition, sensorimotor grounding, ordinality, magnitude, ranking

## Introduction

Number comprehension is critical for being a successful member of society. Recently, Sixtus et al. (2023) proposed a sensorimotor grounding perspective in which number comprehension emerges from the independently grounded concepts of magnitude, ordinality, and cardinality. This perspective integrates the empirical state-of-the-art with core elements from classical theories such as the Triple Code Model and ATOM, rendering an integrative breakthrough in numerical cognition.

A key feature of the perspective by Sixtus et al. (2023) holds that ‘mental lines' underlie ordinality but not magnitude comprehension. This potentially oversimplifies magnitude comprehension. Although we agree that ATOM's original notion of a dedicated mental number line in long-term memory (Walsh, 2003) is not tenable, we argue that an updated notion of ‘mental lines' may still be. Specifically, we propose that both ordinality and magnitude meaning are contextually constructed *in the moment* through referential coding within a spatially scaffolded, low-dimensional *ranking-space* that is itself grounded in sensorimotor experiences. Thus, unless it suffices to merely label an isolated magnitude instance at the cardinal level via counting or estimation (tagging a number-word or Arabic number to it), making sense of a magnitude instance often requires relative magnitude to be determined by placing it in a ranking-space with a contextually appropriate reference point.

Below we first outline the proposed ranking-space through ordinality studies (i.e., explicitly task-relevant ranking), detailing the differences with the conceptual grounding of ordinality proposed by Sixtus et al. (2023). Next, as our core argument against the proposal by Sixtus et al. (2023), we defend an intrinsic involvement of the ranking-space in magnitude comprehension.

## Constructing ordinal meaning

Working with arbitrary (and formally non-spatial) item sequences robustly interacts with (external) spatial processing. Specifically, earlier items in a sequence systematically trigger a relative leftward bias as compared to later items (e.g., Previtali et al., 2010; Van Dijck et al., 2013; Hartmann et al., 2021; Sahan et al., 2021). As such, it is assumed that ordinal context is maintained by spontaneously mapping items onto a mental line in a configuration that reflects the order of items (Abrahamse et al., 2014, 2017; Sixtus et al., 2023).

Here we conceive of the mental line as a low-dimensional *ranking-space* that serves mental coordination for *goal-directed* cognition and behavior, linked to more general literatures on working memory (Oberauer and Hein, 2012; Abrahamse et al., 2017) and conceptual knowledge organization (cf. unidimensional image space; Bottini and Doeller, 2020). The ranking-space is goal-directed due to its flexibility in capturing relevant (hierarchical) relations and its attentional exploitation, as suggested by work on ordinal relations. First, items can be flexibly mapped onto positions of the ranking-space, enabling it to deal with arbitrary or new sequences (e.g., numbers in non-canonical order; Abrahamse et al., 2017). Second, the ranking-space allows to map multiple relevant items – within its capacity limits (e.g., Kreitz et al., 2015; Guida et al., 2017; cf. working memory capacity). The available space can be calibrated to sets of at least 3-5 relevant items (e.g., Van Dijck and Fias, 2011; Van Dijck et al., 2013, 2020). By flexibly calibrating its ‘space' to the relevant item set, contextually meaningful and stable reference points can be established – such as (the item mapped onto) the starting, mid, or endpoint of the ranking-space. These stable reference points enable the maintenance of ordinality via spatial referential coding: The serial position of each item is reflected in its position in the ranking-space *relative* to a specific reference point. Finally, internal spatial attention operates across the ranking-space to select a specific item for focused use. Critically, the latter attentional selection underlies the active construction of *grounded* ordinal meaning during both encoding and retrieval, as the focused item's position in the ranking-space generates a spatial code relative to the reference point – and this spatial code grounds the ordinal meaning (and can interact with processing in external space; Abrahamse et al., 2016, 2017).

The notion that ordinal meaning is grounded by relative position in ranking-space, deviates from the proposal by Sixtus et al. (2023). Whereas they argue for meaning to derive from ‘ordinality concepts' in long-term memory, we propose meaning to be actively constructed in the moment. As such, in contrast to Sixtus et al. (2023), we argue that sensorimotor experiences are *not* grounding conceptual knowledge *per se*, but rather the spatially defined and attentionally exploited scaffolds that afford the construction of meaning.

Specifically, the multi-item ranking-space develops over a mosaic of sensorimotor experiences – potentially starting off from an innate ability to make spatially supported, direct comparisons between two events (e.g., comparison between two current inputs, or between an input and an adapted reference; e.g., De Hevia et al., 2017).^1^ For example, multi-item ordinality increases in relevance when a child learns to count, requiring it to functionally organize more than two items and their specific relations. Here, finger counting allows for cognitive offloading by mapping items (e.g., numbers) onto an external, spatially defined ‘coordination' system (i.e., fingers) that is exploited by external spatial attention. This triggers a hippocampal-parietal network involved in externally oriented spatial processing (cf. Bottini and Doeller, 2020). Over practice, these externally directed procedures are gradually internalized into mental equivalents (i.e., ‘cognitive uploading') that remain supported by the (partially) repurposed hippocampal-parietal network: A mental line serves as an internal coordination system that is operated by internal spatial attention (Abrahamse et al., 2017).

In support of such sensorimotor-driven development, (a) the maintenance and/or retrieval of ordinal information involves the hippocampal-parietal network (Cristoforetti et al., 2022), (b) shifting internal attention across ranking-space induces electrophysiological signatures similar to those obtained from attention shifting in external space (Rasoulzadeh et al., 2021), and (c) the shaping of ranking-space may be functionally affected by early blindness (but see Crollen et al., 2013; Bottini et al., 2016). Finally, once repurposed via sensorimotor experiences such as finger counting, the sensorimotor machinery continues to be shaped by sensorimotor experiences, with ordinal coding increasingly following the dominant reading direction across age (Guida et al., 2018; Van Dijck et al., 2020). Even transient sensorimotor experiences can affect ordinal processing (Guida et al., 2020), serving its goal-directedness.

The development of a dedicated ranking-space may correspond to a gradual transformation of the intraparietal sulcus (IPS). Sensory and motor codes meet in IPS (Sato et al., 2016), including spatial and sensory-magnitude information. Based on ongoing sensorimotor experiences, the IPS may gradually be trained (e.g., through hippocampal and/or prefrontal interactions) into a supramodal, low-dimensional ranking-space that affords goal-directed coding of multiple items and their hierarchical relations. Hence, reminiscent of the role of IPS in spatial cognition for computing object-centered part relations (Ayzenberg and Behrmann, 2022), the IPS involves goal-centered coding of temporarily relevant rankings.

Below, we argue that the IPS-centered ranking-space also underlies the construction of context-specific – and thus relative – magnitude sense. Indeed, this fits findings that the IPS (i) is critical for both ordinal and magnitude information (Fias et al., 2007), (ii) codes for both absolute and relative magnitude or number (Jacob and Nieder, 2009; Mock et al., 2018; Bhatia et al., 2022), and (iii) is modulated by task-relevance in its numerosity-selective responses (Castaldi et al., 2019; Cai et al., 2022).

## Constructing (relative) magnitude meaning

According to Sixtus et al. (2023), “instances of magnitudes can be mentally ordered and mapped onto linear space” (p. 372), but the resulting ordinal relations imply no magnitude relation. However, refuting an intrinsic role of mental lines in magnitude comprehension may be premature. We argue that ranking-space – once developed – is reflexively employed in the construction of context-specific magnitude sense or meaning. This fits with SNARC-like effects obtained from magnitude stimuli that do not provide (explicit) ordinal information (Ren et al., 2011; Fumarola et al., 2014; Macnamara et al., 2018; Richter and Wuehr, 2022; e.g., object size). It can also explain why such effects are sometimes absent when magnitude is task-irrelevant (Cleland et al., 2020), as sensory magnitude cues may be processed but not mapped in the goal-directed ranking-space.

How is magnitude meaning obtained from spatial ranking? To efficiently work with magnitudes and their metrics across different contexts (e.g., dimensions or ranges), internal magnitude codes need to capture *relative* magnitude to be inherently meaningful (Van Opstal and Verguts, 2013; Bonn and Cantlon, 2017). Like ordinality, then, magnitude sense requires a specific reference context. Two proposed candidates for relative magnitude are ratio and rank (Bonn and Cantlon, 2017). We argue that both boil down to relative positions in ranking-space: Spatial ranking can provide the cognitive scaffolds for processing ratios or fractions. For example, maintenance of a fraction (e.g., 2/5) will involve the mapping of the nominator input (2) onto an unidimensional ranking-space (i.e., mental line) that is calibrated to represent denominator range (5). When attentionally focused, the nominator input generates a spatial code in function of its position in the ranking-space, in line with SNARC-like effects obtained from processing fractions (Bonato et al., 2007; Toomarian and Hubbard, 2018) as well as from processing non-symbolic magnitude ratios (Fornaciai et al., 2016; Meng et al., 2019; Wuhr and Richter, 2022). Overall, relative position in ranking-space is the formal code that provides a sense of magnitude.

More generally, we propose that the brain reflexively determines a relative position for each *single* magnitude instance it processes (in a goal-directed manner). Hence, whenever a relevant or highly salient magnitude instance is physically presented in isolation of context, the brain will mentally construct both a ranking-space with a contextually meaningful range, and a reference point from the overall psychological (task) context. The formally isolated input is then placed in the ranking-space based on an interaction between overall task context (top-down) and the physiological excitation that the input's sensory magnitude cues produce (cf. Gebuis et al., 2016). Thus, in itself, such physiological excitation is not equated to meaning as the categorical transformation into sense or meaning requires a relative magnitude to be constructed; yet, sense or meaning may evolve very rapidly if the referent (top-down component) is already attentionally established to bias bottom-up processing of a new input (cf. Castaldi et al., 2021).

So, what is the reference point for a single magnitude instance? In some cases, the overall relevant item set will determine the reference point. For example, when presenting objects of different sizes across trials, the average size across them is learnt and then mapped in ranking-space, serving as the reference point against which each new input is compared in order to form relative magnitude senses (e.g., Wuhr and Richter, 2022). In other cases, the reference point may derive from previous stimuli. For example, when sequentially presenting participants with two different-sized stimuli, the second stimulus will generate a magnitude sense relative to the first (Ren et al., 2011). Indeed, a similar logic can be applied to numerosity studies that use explicit adapter stimuli (e.g., Fornaciai et al., 2016), suggesting that non-symbolic numerosity sense behaves like other magnitude dimensions (Dehaene, 2003; Anobile et al., 2021).

Reference points may also derive from outside the explicit task set. For example, an ink stain on a computer screen may obtain a sense of relative magnitude in reference to the overall screen size, and an animal may make rapid estimation of the relative number of enemies or food sources in an environment by comparing it to a running average that the animal has acquired across life (cf. Cicchini et al., 2016). Such involvement of general experience can also be seen in humans, such as with SNARC-like effects by task-irrelevant pitch in musicians but not in non-musicians (Cho et al., 2012).

Finally, magnitude sense obtained via spatial ranking can also account for the type of cross-domain interactions that are typically considered as support for a generalized magnitude system. When multiple ordinal and/or magnitude dimensions are relevant (or saliently present), parallel lines may be formed within the overall ranking-space – one for each dimension (cf. Sheahan et al., 2021). When these parallel lines are normalized for comparing different dimensions to each other (Sheahan et al., 2021), cross-dimension interactions may follow because attention shifts along one mental line affect the processing of information on a parallel line. Overall, then, ranking-space may not be incompatible with the notion of a generalized magnitude system, even more when considering spatial distance to a reference point to approximate a prothetic dimension (cf. Walsh, 2003).^2^

## Conclusion

We propose a goal-directed ranking-space centered on the IPS to underlie the construction of both ordinal and (relative) magnitude meaning. Whether a task emphasizes ordinal or magnitude information, ultimately both need ranking-space to construct categorical decisions about contextually relative meaning (e.g., is the input more or less, small or large, closer to 1 or to 5, 1st or 3rd?). As such, number comprehension may not evolve from sensorimotor grounded concepts (Sixtus et al., 2023) but be contextually constructed in the moment via spatial scaffolds that develop and shape through sensorimotor experiences (much like has been proposed for other abstract concepts; Kintsch and Mangalath, 2011). Future studies should empirically scrutinize our proposal and its relation to the perspective of Sixtus et al. (2023) as either complementary or competitor accounts.

## Author contributions

EA and J-PvD contributed to the conceptualization of the paper. EA wrote the first draft of the manuscript. All authors contributed to manuscript revision, read, and approved the submitted version.

## Footnotes

^1^An ability potentially shared with animals (Rugani et al., 2015).

^2^For a related discussion, see Pitt and Casasanto (2019, 2022) vs. Prpic et al. (2021).

## References

[B1] Abrahamse E. Van Dijck J. P. Fias W. (2016). How does working memory enable number-induced spatial biases? Front. Psychol. 7, 977. 10.3389/fpsyg.2016.00977 27445937 PMC4925657

[B2] Abrahamse E. Van Dijck J. P. Majerus S. Fias W. (2014). Finding the answer in space: the mental whiteboard hypothesis on serial order in working memory. Front Hum. Neurosci. 8, 932. 10.3389/fnhum.2014.00932 25505394 PMC4243569

[B3] Abrahamse E. L. van Dijck J. P. Fias W. (2017). Grounding verbal working memory: the case of serial order. Curr. Directions n Psychol. Sci. 26, 429–433. 10.1177/0963721417704404

[B4] Anobile G. Arrighi R. Castaldi E. Burr D. C. (2021). A sensorimotor numerosity system. Trends Cognit. Sci. 25, 24–36. 10.1016/j.tics.2020.10.009 33221159

[B5] Ayzenberg V. Behrmann M. (2022). The dorsal visual pathway represents object-centered spatial relations for object recognition. J. Neurosci. 42, 4693–4710. 10.1523/JNEUROSCI.2257-21.2022 35508386 PMC9186804

[B6] Bhatia P. Longo L. Chesnokova H. Prado J. (2022). Neural representations of absolute and relative magnitudes in symbolic and nonsymbolic formats. Cereb. Cortex 32, 4733–4745. 10.1093/cercor/bhab513 35134134

[B7] Bonato M. Fabbri S. Umilt,à C. Zorzi M. (2007). The mental representation of numerical fractions: Real or integer?. J. Exp. Psychol. Hum. Percep. Performance 33, 1410. 10.1037/0096-1523.33.6.1410 18085953

[B8] Bonn C. D. Cantlon J. F. (2017). Spontaneous, modality-general abstraction of a ratio scale. Cognition 169, 36–45. 10.1016/j.cognition.2017.07.012 28806722 PMC5636217

[B9] Bottini R. Doeller C. F. (2020). Knowledge across reference frames: Cognitive maps and image spaces. Trends Cognit. Sci. 24, 606–619. 10.1016/j.tics.2020.05.008 32586649

[B10] Bottini R. Mattioni S. Collignon O. (2016). Early blindness alters the spatial organization of verbal working memory. Cortex 83, 271–279. 10.1016/j.cortex.2016.08.007 27622641

[B11] Cai Y. Hofstetter S. Harvey B. M. Dumoulin S. O. (2022). Attention drives human numerosity-selective responses. Cell Rep. 39, 111005. 10.1016/j.celrep.2022.111005 35767956

[B12] Casasanto D. Pitt B. (2019). The faulty magnitude detector: Why SNARC-like tasks cannot support a generalized magnitude system. Cognitive Sci. 43, e12794. 10.1111/cogs.12794 31621122

[B13] Castaldi E. Piazza M. Dehaene S. Vignaud A. Eger E. (2019). Attentional amplification of neural codes for number independent of other quantities along the dorsal visual stream. Elife 8, e45160. 10.7554/eLife.45160.024 31339490 PMC6693892

[B14] Castaldi E. Pom,è A. Cicchini G. M. Burr D. Binda P. (2021). The pupil responds spontaneously to perceived numerosity. Nature Commun. 12, 5944. 10.1038/s41467-021-26261-4 34642335 PMC8511033

[B15] Cho Y. S. Bae G. Y. Proctor R. W. (2012). Referential coding contributes to the horizontal SMARC effect. J. Exp. Psychol. Hum. Percep. Perf. 38, 726. 10.1037/a0026157 22141586

[B16] Cicchini G. M. Anobile G. Burr D. C. (2016). Spontaneous perception of numerosity in humans. Nat. Commun. 7, 12536. 10.1038/ncomms12536 27555562 PMC4999503

[B17] Cleland A. A. Corsico K. White K. Bull R. (2020). Non-symbolic numerosities do not automatically activate spatial–numerical associations: Evidence from the SNARC effect. Q. J. Exp. Psychol. 73, 295–308. 10.1177/1747021819875021 31432745

[B18] Cristoforetti G. Majerus S. Sahan M. I. Van Dijck J. P. Fias W. (2022). Neural patterns in parietal cortex and hippocampus distinguish retrieval of start versus end positions in working memory. J. Cognit. Neurosci. 34, 1230–1245. 10.1162/jocn_a_01860 35556132

[B19] Crollen V. Dormal G. Seron X. Lepore F. Collignon O. (2013). Embodied numbers: the role of vision in the development of number–space interactions. Cortex 49, 276–283. 10.1016/j.cortex.2011.11.006 22178125

[B20] De Hevia D. Veggiotti M. D. Streri L. A. Bonn C. D. (2017). At birth, humans associate “few” with left and “many” with right. Current Biol. 27, 3879–3884. 10.1016/j.cub.2017.11.024 29225024

[B21] Dehaene S. (2003). The neural basis of the Weber–Fechner law: a logarithmic mental number line. Trends Cognitive Sci. 7, 145–147. 10.1016/S1364-6613(03)00055-X 12691758

[B22] Fias W. Lammertyn J. Caessens B. Orban G. A. (2007). Processing of abstract ordinal knowledge in the horizontal segment of the intraparietal sulcus. J. Neurosci. 27, 8952–8956. 10.1523/JNEUROSCI.2076-07.2007 17699676 PMC6672167

[B23] Fornaciai M. Cicchini G. M. Burr D. C. (2016). Adaptation to number operates on perceived rather than physical numerosity. Cognition 151, 63–67. 10.1016/j.cognition.2016.03.006 26986745 PMC5040501

[B24] Fumarola A. Prpic V. Pos D. A. Murgia O. Umiltà M. C. Agostini T. (2014). Automatic spatial association for luminance. Attent. Percep. Psychophys. 76, 759–765. 10.3758/s13414-013-0614-y 24402699

[B25] Gebuis T. Kadosh R. C. Gevers W. (2016). Sensory-integration system rather than approximate number system underlies numerosity processing: A critical review. Acta Psychol. 171, 17–35. 10.1016/j.actpsy.2016.09.003 27640140

[B26] Guida A. Abrahamse E. van Dijck J. P. (2020). About the interplay between internal and external spatial codes in the mind: implications for serial order. Annal. Acad. Sci. 1477, 20–33. 10.1111/nyas.14341 32314419

[B27] Guida A. Megreya A. M. Lavielle-Guida M. Noël Y. Mathy F. van Dijck J. P.. (2018). Spatialization in working memory is related to literacy and reading direction: culture “literarily” directs our thoughts. Cognition 175, 96–100. 10.1016/j.cognition.2018.02.013 29486378

[B28] Guida A. van Dijck J. P. Abrahamse E. (2017). Distinctiveness as a function of spatial expansion in verbal working memory: comment on Kreitz, Furley, Memmert, and Simons (2015). Psychol. Res. 81, 690–695. 10.1007/s00426-016-0765-2 27000048

[B29] Hartmann M. Martarelli C. S. Sommer N. R. (2021). Early is left and up: Saccadic responses reveal horizontal and vertical spatial associations of serial order in working memory. Cognit.ion 217, 104908. 10.1016/j.cognition.2021.104908 34543935

[B30] Jacob S. N. Nieder A. (2009). Notation-independent representation of fractions in the human parietal cortex. J. Neurosci. 29, 4652–4657. 10.1523/JNEUROSCI.0651-09.2009 19357289 PMC6665727

[B31] Kintsch W. Mangalath P. (2011). The construction of meaning. Topics Cognit. Sci. 3, 346–370. 10.1111/j.1756-8765.2010.01107.x 25164299

[B32] Kreitz C. Furley P. Memmert D. Simons D. J. (2015). Working-memory performance is related to spatial breadth of attention. Psychological Research 79, 1034–1041. 10.1007/s00426-014-0633-x 25468209

[B33] Macnamara A. Keage H. A. Loetscher T. (2018). Mapping of non-numerical domains on space: a systematic review and meta-analysis. Exp. Brain Res. 236, 335–346. 10.1007/s00221-017-5154-6 29279982

[B34] Meng R. Matthews P. G. Toomarian E. Y. (2019). The relational SNARC: Spatial representation of nonsymbolic ratios. Cognit. Sci. 43, e12778. 10.1111/cogs.12778 31446660

[B35] Mock J. Huber S. Bloechle J. Dietrich J. F. Bahnmueller J. Rennig J.. (2018). Magnitude processing of symbolic and non-symbolic proportions: an fMRI study. Behav. Brain Functions 14, 1–19. 10.1186/s12993-018-0141-z 29747668 PMC5944011

[B36] Oberauer K. Hein L. (2012). Attention to information in working memory. Curr. Directions Psychol. Sci. 21, 164–169. 10.1177/0963721412444727

[B37] Pitt B. Casasanto D. (2022). The order of magnitude: why snarc-like tasks (still) cannot support a generalized magnitude system. Cognit. Sci. 46, e13108. 10.1111/cogs.13108 35174896

[B38] Previtali P. Hevia D. Girelli M. D. (2010). Placing order in space: The SNARC effect in serial learning. Exp. Brain Res. 201, 599–605. 10.1007/s00221-009-2063-3 19888566

[B39] Prpic V. Mingolo S. Agostini T. Murgia M. (2021). Magnitude and order are both relevant in SNARC and SNARC-like effects: a commentary on Casasanto and Pitt (2019). Cognit. Sci. 45, e13006. 10.1111/cogs.13006 34213789

[B40] Rasoulzadeh V. Sahan M. I. van Dijck J. P. Abrahamse E. Marzecova A. Verguts T.. (2021). Spatial attention in serial order working memory: An EEG study. Cereb. Cortex 31, 2482–2493. 10.1093/cercor/bhaa368 33305807

[B41] Ren P. Nicholls M. E. Ma Y. Y. Chen L. (2011). Size matters: Non-numerical magnitude affects the spatial coding of response. PLoS ONE 6, e23553. 10.1371/journal.pone.0023553 21853151 PMC3154948

[B42] Richter M. Wuehr P. (2022). The nature of associations between physical stimulus size and left-right response codes. J. Cognit. 5, 15. 10.5334/joc.206 36072110 PMC9400664

[B43] Rugani R. Vallortigara G. Priftis K. Regolin L. (2015). Number-space mapping in the newborn chick resembles humans' mental number line. Sci.ence 347, 534–536. 10.1126/science.aaa1379 25635096

[B44] Sahan M. I. van Dijck J. P. Fias W. (2021). Eye-movements reveal the serial position of the attended item in verbal working memory. Psychonomic Bullet. Rev. 29, 530–540. 10.3758/s13423-021-02005-9 34582030

[B45] Sato J. R. Biazoli C. E. Jr. Salum G. A. Gadelha A. Crossley N. Vieira G.. (2016). Connectome hubs at resting state in children and adolescents: Reproducibility and psychopathological correlation. Dev. Cognit. Neurosci. 20, 2–11. 10.1016/j.dcn.2016.05.002 27288820 PMC6987719

[B46] Sheahan H. Luyckx F. Nelli S. Teupe C. Summerfield C. (2021). Neural state space alignment for magnitude generalization in humans and recurrent networks. Neuron 109, 1214–1226. 10.1016/j.neuron.2021.02.004 33626322

[B47] Sixtus E. Krause F. Lindemann O. Fischer M. H. (2023). A sensorimotor perspective on numerical cognition. Trends Cognit. Sci. 23, 1–5. 10.1016/j.tics.2023.01.002 36764902

[B48] Toomarian E. Y. Hubbard E. M. (2018). The fractions SNARC revisited: processing fractions on a consistent mental number line. Quarterly J. Exp. Psychol. 71, 1761–1770. 10.1080/17470218.2017.1350867 28697687

[B49] Van Dijck J. P. Abrahamse E. Fias W. (2020). Do preliterate children spontaneously employ spatial coding for serial order in working memory?. Annals Acad. Sci. 1477, 91–99. 10.1111/nyas.14430 32761632

[B50] Van Dijck J. P. Abrahamse E. L. Majerus S. Fias W. (2013). Spatial attention interacts with serial-order retrieval from verbal working memory. Psychol. Sci. 24, 1854–1859. 10.1177/0956797613479610 23863755

[B51] Van Dijck J. P. Fias W. (2011). A working memory account for spatial–numerical associations. Cognition 119, 114–119. 10.1016/j.cognition.2010.12.013 21262509

[B52] Van Opstal F. Verguts T. (2013). Is there a generalized magnitude system in the brain? Behavioral, neuroimaging, and computational evidence. Front. Psychol. 4, 435. 10.3389/fpsyg.2013.00435 23874319 PMC3711208

[B53] Walsh V. (2003). A theory of magnitude: common cortical metrics of time, space and quantity. Trends Cogniti. Sci. 7, 483–488. 10.1016/j.tics.2003.09.002 14585444

[B54] Wuhr P. Richter M. (2022). Relative, not absolute, stimulus size is responsible for a correspondence effect between physical stimulus size and left/right responses. Attent. Percep. Psychophys. 84, 1342–1358. 10.3758/s13414-022-02490-7 35460026 PMC9032296

